# An Efficient Nomogram for Discriminating Intrahepatic Cholangiocarcinoma From Hepatocellular Carcinoma: A Retrospective Study

**DOI:** 10.3389/fonc.2022.833999

**Published:** 2022-04-11

**Authors:** Yuan-Quan Si, Xiu-Qin Wang, Cui-Cui Pan, Yong Wang, Zhi-Ming Lu

**Affiliations:** ^1^ Department of Clinical Laboratory, Shandong Provincial Hospital Affiliated to Shandong First Medical University, Jinan, China; ^2^ School of Basic Medicine, Shandong First Medical University, Jinan, China

**Keywords:** intrahepatic cholangiocarcinoma, hepatocellular carcinoma, nomogram, alpha-fetoprotein, PIVKA-II, CA199, CA125

## Abstract

**Objective:**

This study aims to establish a nomogram and provide an effective method to distinguish between intrahepatic cholangiocarcinoma (ICC) and hepatocellular carcinoma (HCC).

**Methods:**

A total of 1,591 patients with HCC or ICC hospitalized at Shandong Provincial Hospital between January 2016 and August 2021 were included and randomly divided into development and validation groups in a ratio of 3:1. Univariate and multivariate analyses were performed to determine the independent differential factors between HCC and ICC patients in the development cohort. By combining these independent differential factors, the nomogram was established for discriminating ICC from HCC. The accuracy of the nomogram was estimated by using receiver operating characteristic (ROC) curve and decision curve analysis (DCA). Furthermore, the predictive nomogram was assessed in the internal testing set.

**Results:**

Through multivariate analysis, independent differential factors between HCC and ICC involved hepatitis B virus (HBV), logarithm of alpha-fetoprotein (Log AFP), logarithm of protein induced by vitamin K absence or antagonist-II (Log PIVKA-II), logarithm of carbohydrate antigen 199 (Log CA199), and logarithm of carbohydrate antigen 125 (Log CA125). A nomogram was finally established by incorporating these five independent differential factors. Comparing a model of conventional tumor biomarkers including AFP and CA199, the nomogram showed a better distinction between ICC and HCC. The area under the ROC curve (AUC) of ICC diagnosis was 0.951 (95% CI, 0.938–0.964) for the nomogram. The results were consistent in the validation cohort with an AUC of 0.958 (95% CI, 0.938–0.978). After integrating patient preferences into the analysis, the DCA showed that using this nomogram to distinguish ICC and HCC increased more benefit compared with the conventional model.

**Conclusion:**

An efficient nomogram has been established for the differential diagnosis between ICC and HCC, which may facilitate the detection and diagnosis of ICC. Further use of the nomogram in multicenter investigations will confirm the practicality of the tool for future clinical application.

## Introduction

Primary liver cancer (PLC) is the sixth most common cancer and the third leading cause of cancer-related death worldwide in 2020, which includes HCC and ICC, and mixed hepatocellular–cholangiocarcinoma carcinoma according to different cell origin (1). Among them, ICC derived from the epithelial cell of intrahepatic bile duct is the second most common liver malignancy (2). Although ICC is not as common as HCC, its incidence rate has risen sharply in recent years without clear etiology (3, 4).

However, due to the different molecular mechanisms of carcinogenesis, the survival and prognosis of ICC are worse than HCC. Despite recent advances in basic research and clinical trials, it is reported that the 5-year survival rate of ICC is only about 30%. As a malignant neoplasm, ICC often shares some common hazard factors and clinical features with HCC, which is a challenge for the differential diagnosis of ICC and HCC. With this in mind, it is urgent to find an effective and specific method that can provide early prediction value for the differential diagnosis of ICC and HCC (5–7).

The gold standard of differential diagnosis between ICC and HCC relies on pathological examination yet in the current clinical practice (4). The two tumor markers (AFP and CA199) are the most commonly used cancer biomarkers to distinguish ICC and HCC. However, the diagnostic specificity and sensitivity of these biomarkers are still not satisfactory (8–11). Although lots of research have been carried out to study the characteristics of ICC and seek new differential diagnostic markers to distinguish ICC from HCC, their effects in clinical application still remain weak (12, 13). In view of the lack of highly specific and sensitive predictive biomarkers to diagnose ICC, the establishment of predictive models including independent factors may be a feasible way to resolve the issue. Nomograms are recently described as simple graphical systems, which may be more accurate than traditional methods in preoperative diagnosis and prognostic evaluation for a variety of malignant tumors, including liver cancer and pancreatic adenocarcinoma (14–16). In order to distinguish between ICC and HCC before surgery without pathological verification, our study is to establish and verify the nomogram model for the differential diagnosis of ICC and HCC based on demographic characteristics and the results of routine laboratory tests.

## Materials and Methods

### Patients

**Figure 1:**
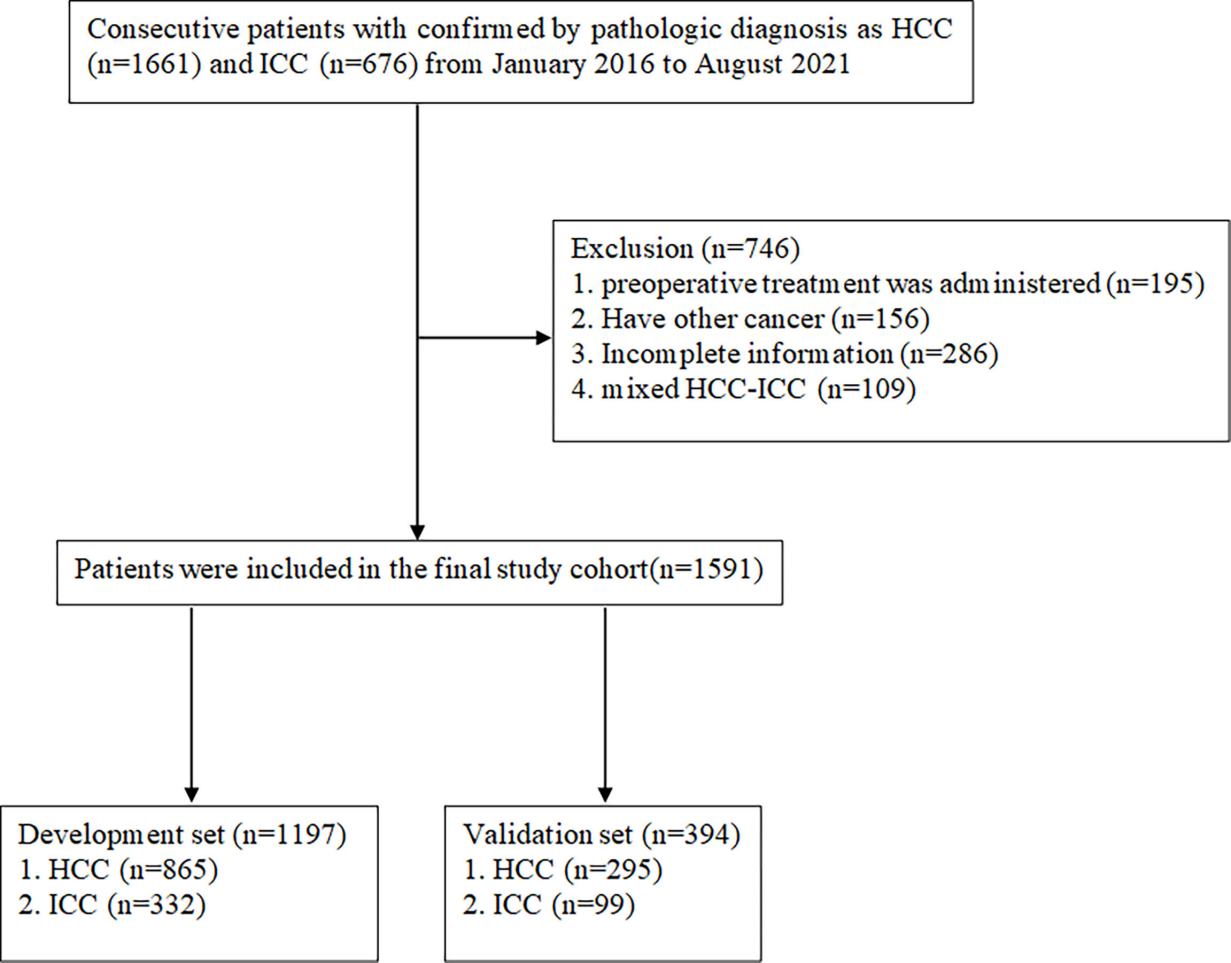
Flowchart detailing the patient selection process and exclusion criteria.

A total of 1,591 patients with HCC or ICC who received curative surgery for PLC at Shandong Provincial Hospital between January 2016 and August 2021 were included in this retrospective study. The patients were selected according to the inclusion and elimination criteria, just as shown in **Figure 1**. The inclusion criteria entailed pathologically confirmed HCC or ICC patients over the age of 18. The reasons for exclusion were as follows: (1) incomplete clinical information; (2) mixed hepatocellular–cholangiocellular carcinoma or other types of liver tumor; (3) with medical history of other cancers; (4) with preoperative treatments. Patients who met the inclusion and exclusion criteria were divided into development and validation groups randomly in a ratio of 3:1. All procedures involving human participants have been approved by the Shandong Provincial Hospital Research Ethics Committee. The data were anonymous, and the requirement for informed consent was therefore waived (17).

### Clinicopathological Variables

Demographic variables including age, gender, hepatitis B history, hepatitis C history and clinicopathological staging of ICC and HCC were obtained. Serum examination included AFP, AFP-L3%, PIVKA-II, carcinoembryonic antigen (CEA), CA199, CA125, alanine transaminase (ALT), aspartate transaminase (AST), γ-glutamyl transpeptidase (GGT), total bilirubin (TBIL), direct bilirubin (DBIL), total protein (TP), albumin (ALB), red blood cell (RBC), white blood cell (WBC), and platelet (PLT). In our study, the contents of these tests were detected respectively before undergoing scheduled surgery. AFP and AFP-L3 were detected by the method of immunofluorescence on automatic electrophoresis fluorescence immunoassay instrument (mTAS Wako i30, Japan). AFP-L3% was measured by the AFP-L3 divided by the AFP. PIVKA-II was measured using chemiluminescence enzyme immunoassay on automatic chemiluminescence immunoassay analyzer (LUMIPULSE^®^G1200, Japan). Detection of CEA, CA199, and CA125 was performed using an automatic electrochemiluminescence analyzer (Roche Diagnostics, Mannheim, Germany). Serum levels of ALT, AST, GGT, TP, ALB, TBIL, and DBIL were analyzed using automatic biochemical analyzer (AU5831, USA). The values of PLT, RBC and WBC were measured with fully automated hematology analyzer (Sysmex XN-9000, Sysmex, Kobe, Japan). The above reagents required were all original kits, and the tests were carried out in strict accordance with the standard operation procedures.

### Statistical Analysis

Numerical variables were expressed as mean with standard deviation (SD) or median with interquartile range (IQR), which were compared by Student’s *t*-test or Mann–Whitney test, respectively. Categorical variables were expressed as frequencies and compared using Pearson’s χ^2^ test or Fisher exact test. Log transformation was performed for these variables with skewed distributions such as AFP, AFP-L3%, PIVKA-II, CEA, CA199, CA125, ALT, AST, GGT, TBIL, and DBIL. Univariate and multivariate regression analyses were adopted to determine the independent differential factors between ICC and HCC. A nomogram was drawn according to these screened independent difference factors, and the total score of each patient was calculated using this established nomogram. The diagnostic ability of our nomogram was estimated by the ROC curve and AUC. Then, Z-test was applied to compare the difference between our nomogram and other model. The decision curve analysis (DCA) was used to assess the clinical utility value of the nomogram and other model by quantifying net benefits against a range of threshold probabilities (18). We further evaluated whether the use of indicator variables with missing data biased our results by performing multivariate multiple imputation analysis. We repeated all analyses with the complete data (19–21). The nomogram and DCA were established with R (http://www.R-project.org) and EmpowerStats software (www.empowerstats.com, Boston MA, USA) (22). Other analyses were performed by IBM SPSS software (version 25.0, USA) and MedCalc (version 20.0.8, Belgium). The *p*<0.05 was considered statistically significant.

## Results

### Patient Demographics and Clinicopathologic Variables

During the study period, a total of 1,591 consecutive patients who underwent hepatectomy for primary hepatic carcinoma and met the inclusion criteria were enrolled. Among them, 1,197 and 394 patients formed the training and validation cohort, respectively. In the training group, 865 HCC patients and 332 ICC patients were included. The validation cohort consisted of 295 HCC patients and 99 ICC patients. The demographics and clinicopathological variables of the training and validation cohort patients are listed in **Table 1**. A comparison of baseline data showed that there were no significant differences in general conditions and other indicators between the former two cohorts. Meanwhile, the baseline clinicopathological data were compared between ICC and HCC of training cohort (**Table 2**).

**Table 1:** Baseline characteristics of the development and validation groups.

Variables	Training (n = 1197)	Validation (n = 394)	*p*-value
Age (years)	57.82 (10.36)	57.77 (10.12)	0.942
Sex			0.642
Male	931 (77.78%)	302 (76.65%)	
Female	266 (22.22%)	92 (23.35%)	
PLT (10^9^/L)	190.19 (91.19)	185.18 (86.14)	0.338
RBC (10^12^/L)	4.42 (0.62)	4.38 (0.66)	0.252
WBC (10^9^/L)	5.99 (2.75)	5.86 (2.63)	0.391
AFP (ng/mL)	9.20 (2.70–181.50)	8.94 (2.90–195.05)	0.575
AFP-L3%	4.60 (0.50–32.40)	3.50 (0.50–19.98)	0.143
CEA (ng/ml)	2.83 (1.80–4.57)	2.83 (1.88–4.30)	0.837
CA125 (U/ml)	14.80 (9.71–29.43)	13.56 (8.91–28.19)	0.099
CA199 (IU/ml)	21.20 (11.94–56.00)	21.45 (11.57–58.75)	0.851
PIVKA-II (mAU/ml)	114.00 (27.78–1266.06)	146.92 (28.25–1776.50)	0.374
AST (U/L)	33.00 (24.00–55.00)	37.00 (25.00–60.75)	0.036
ALT (U/L)	29.00 (19.00–51.00)	32.50 (20.00–53.00)	0.212
TP (g/L)	69.66 (7.25)	70.01 (7.28)	0.411
ALB (g/L)	39.89 (5.31)	39.47 (5.24)	0.181
GGT (U/L)	55.00 (29.00–131.00)	60.00 (32.00–148.50)	0.083
TBIL (μmol/L)	16.50 (12.34–23.56)	16.69 (12.00–22.38)	0.721
DBIL (μmol/L)	3.64 (2.60–5.71)	3.70 (2.52–5.65)	0.727
HBV			0.448
No	383 (32.00%)	118 (29.95%)	
Yes	814 (68.00%)	276 (70.05%)	
HCV			0.126
No	1,166 (97.41%)	389 (98.73%)	
Yes	31 (2.59%)	5 (1.27%)	
Clinicopathological staging			0.648
Well differentiation	176 (14.70%)	56 (14.21%)	
Moderate differentiation	720 (60.15%)	247 (62.69%)	
Poor differentiation	301 (25.15%)	91 (23.10%)	

Categorical variables are expressed as frequency. Continuous variables are expressed as mean (SD) or median with interquartile range (IQR).

PLT, platelet; RBC, red blood cell; WBC, white blood cell; AFP, α-fetoprotein level; AFP-L3, an isoform of AFP characterized by the presence of an a 1–6-linked residue on the AFP carbohydrate side chain; CA199, carbohydrate antigen 199; CEA, carcinoembryonic antigen; PIVKA-II, protein induced by vitamin K absence or antagonist-II; CA125, carbohydrate antigen 125; ALT, alanine transaminase; AST, aspartate transaminase; GGT, gamma glutamyl transpeptidase; TP, total protein; ALB, albumin; TBIL, total bilirubin; DBIL, direct bilirubin; HBV, hepatitis B virus; HCV, hepatitis C virus.

**Table 2:** Demographic information and clinicopathological characteristics of the training cohort.

Variables	HCC (n=865)	ICC (n=332)	*p*-value
Age (years)	57.15 (10.19)	59.56 (10.59)	<0.001
Sex			<0.001
Male	718 (83.01%)	213 (64.16%)	
Female	147 (16.99%)	119 (35.84%)	
PLT (10^9^/L)	170.98 (84.26)	240.22 (89.68)	<0.001
RBC (10^12^/L)	4.46 (0.61)	4.33 (0.63)	0.001
WBC (10^9^/L)	5.54 (2.54)	7.17 (2.92)	<0.001
AFP (ng/ml)	35.90 (4.20–558.10)	2.69 (1.87–4.43)	<0.001
AFP–L3%	10.00 (0.50–38.70)	0.50 (0.50–0.50)	<0.001
CEA (ng/ml)	2.50 (1.69–3.88)	3.90 (2.13–11.55)	<0.001
CA125 (U/ml)	13.04 (9.09–22.27)	23.90 (12.48–80.55)	<0.001
CA199 (IU/ml)	17.16 (10.90–29.48)	125.00 (23.91-1000.00)	<0.001
PIVKA-II (mAU/ml)	367.01 (49.08–2839.86)	27.66 (20.77–42.09)	<0.001
AST (U/L)	34.00 (25.00–53.00)	31.00 (22.00–61.00)	0.133
ALT (U/L)	30.00 (20.00–49.00)	27.00 (16.75–63.00)	0.138
TP (g/L)	69.87 (7.11)	69.12 (7.57)	0.106
ALB (g/L)	40.05 (5.32)	39.47 (5.25)	0.092
GGT (U/L)	49.00 (27.00–102.00)	87.50 (37.75–268.75)	<0.001
TBIL (μmol/L)	16.40 (12.47–22.60)	16.93 (12.00–36.61)	0.013
DBIL (μmol/L)	3.67 (2.67–5.44)	3.60 (2.50–12.12)	0.026
HBV			<0.001
No	123 (14.22%)	260 (78.31%)	
Yes	742 (85.78%)	72 (21.69%)	
HCV			0.144
No	839 (96.99%)	327 (98.49%)	
Yes	26 (3.01%)	5 (1.51%)	
Clinicopathological staging			0.009
Well differentiation	114 (13.18%)	62 (18.67%)	
Moderate differentiation	542 (62.66%)	178 (53.61%)	
Poor differentiation	209 (24.16%)	92 (27.71%)	

Categorical variables are expressed as frequency. Continuous variables are expressed as mean (SD) or median with interquartile range (IQR).

PLT, platelet; RBC, red blood cell; WBC, white blood cell; AFP, α-fetoprotein level; AFP-L3, an isoform of AFP characterized by the presence of an a 1–6-linked residue on the AFP carbohydrate side chain; CA199, carbohydrate antigen 199; CEA, carcinoembryonic antigen; PIVKA-II, protein induced by vitamin K absence or antagonist-II; CA125, carbohydrate antigen 125; ALT, alanine transaminase; AST, aspartate transaminase; GGT, gamma glutamyl transpeptidase; TP, total protein; ALB, albumin; TBIL, total bilirubin; DBIL, direct bilirubin; HBV, hepatitis B virus; HCV, hepatitis C virus.

### Univariate and Multivariate Analysis of Independent Differences Between ICC and HCC Patients

As shown in **Table 3**, the univariate analysis of training cohort indicated that except for a few indicators (such as Log ALT, Log AST, TP, and ALB), all other indicators were potential difference factors between ICC and HCC patients (*p* < 0.01). All these potential difference factors were then brought into multivariate logistic regression. Only Log AFP (OR = 0.46; 95% CI, 0.31–0.68, *p* = 0.0001), Log PIVKA-II (OR = 0.19; 95% CI, 0.12–0.29, *p*< 0.0001), Log CA199 (OR = 2.88; 95% CI, 1.95–4.26, *p*< 0.0001), Log CA125 (OR = 2.75; 95% CI, 1.5–5.01, *p* = 0.001), and HBV (OR = 0.13; 95% CI, 0.08–0.22, *p*< 0.0001) were the independent difference for the presence of ICC and HCC.

**Table 3:** Univariate and multivariate logistic regression analysis of ICC presence based on preoperative data in training cohort.

Variables	Univariable		Multivariable	
	OR (95%CI)	*p*-value	OR (95%CI)	*p*-value
Age (years)	1.02 (1.01, 1.04)	0.003	NA	
Sex		<0.0001		
Male	reference		reference	
Female	2.73 (2.05, 3.63)		NA	
PLT (10^9^/L)	1.01 (1.01, 1.01)	<0.0001	NA	
RBC (10^12^/L)	0.72 (0.58, 0.88)	0.0012	NA	
WBC (10^9^/L)	1.24 (1.18, 1.31)	<0.0001	NA	
Log AFP (ng/ml)	0.24 (0.19, 0.30)	<0.0001	0.47 (0.32, 0.69)	0.0001
Log AFP-L3%	0.35 (0.30, 0.42)	<0.0001	NA	
Log CEA (ng/ml)	5.46 (3.92, 7.62)	<0.0001	NA	
Log CA125 (U/ml)	4.35 (3.28, 5.77)	<0.0001	2.74 (1.50, 5.01)	0.001
Log CA199 (IU/ml)	7.68 (5.90, 9.98)	<0.0001	2.88 (1.95, 4.25)	<0.0001
Log PIVKA-II (mAU/ml)	0.20 (0.16, 0.26)	<0.0001	0.19 (0.13, 0.30)	<0.0001
Log AST (U/L)	1.02 (0.69, 1.53)	0.9105	NA	
Log ALT (U/L)	1.12 (0.80, 1.57)	0.5168	NA	
TP (g/L)	0.99 (0.97, 1.00)	0.106	NA	
ALB (g/L)	0.98 (0.96, 1.00)	0.0927	NA	
Log GGT (U/L)	3.32 (2.53, 4.36)	<0.0001	NA	
Log TBIL (μmol/L)	3.69 (2.60, 5.24)	<0.0001	NA	
Log DBIL (μmol/L)	2.82 (2.17, 3.66)	<0.0001	NA	
HBV		<0.0001		<0.0001
No	reference		reference	
Yes	0.05 (0.03, 0.06)		0.13 (0.08, 0.22)	
HCV		0.1516		
No	reference		reference	
Yes	0.49 (0.19, 1.30)		NA	

Categorical variables are expressed as frequency. Continuous variables are expressed as mean (SD) or median with interquartile range (IQR).

PLT, platelet; RBC, red blood cell; WBC, white blood cell; AFP, α-fetoprotein level; AFP-L3, an isoform of AFP characterized by the presence of an a 1–6-linked residue on the AFP carbohydrate side chain; CA199, carbohydrate antigen 199; CEA, carcinoembryonic antigen; PIVKA-II, protein induced by vitamin K absence or antagonist-II; CA125, carbohydrate antigen 125; ALT, alanine transaminase; AST, aspartate transaminase; GGT, gamma glutamyl transpeptidase; TP, total protein; ALB, albumin; TBIL, total bilirubin; DBIL, direct bilirubin; HBV, hepatitis B virus; HCV, hepatitis C virus; NA, Not applicable.

### Development and Validation of a Nomogram for the Differential Diagnosis Between ICC and HCC

**Figure 2:**
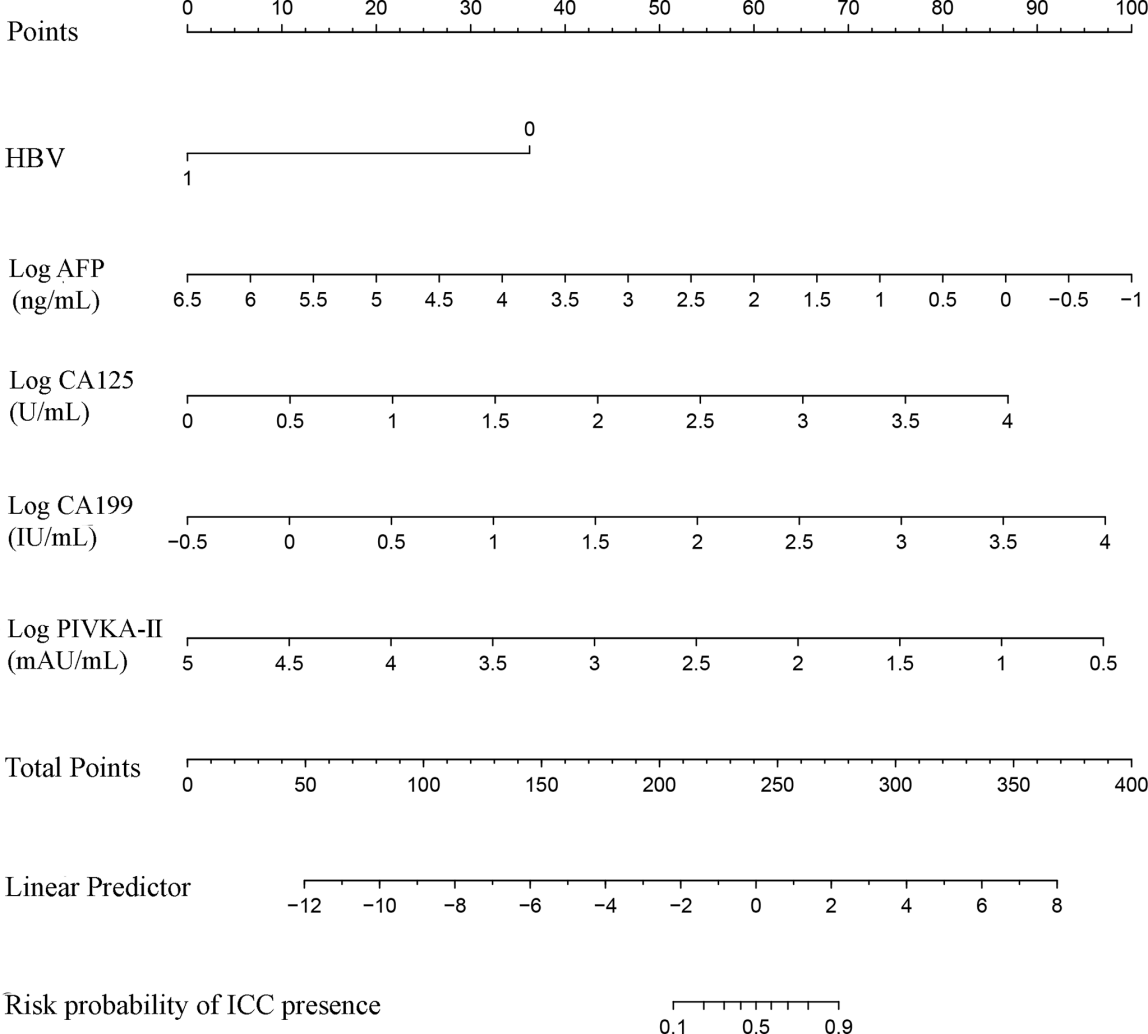
The nomogram discriminates ICC from HCC. To use the nomogram, find the position of each variable on the corresponding axis, then draw a line to the points axis at the top of the nomogram to calculate the respective points for each parameter; finally, add the total points from all parameters and draw a line from the total points axis to the risk probability axis at the bottom of the nomogram to determine ICC presence probabilities.

**Figure 3:**
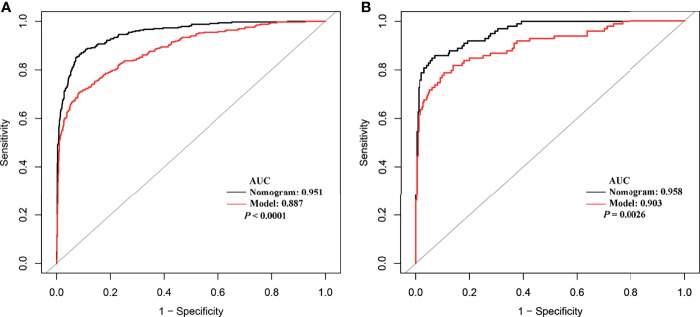
The receiver operating characteristic curves of the nomogram and model from development cohort **(A)** and validation cohort **(B)**.

**Figure 4:**
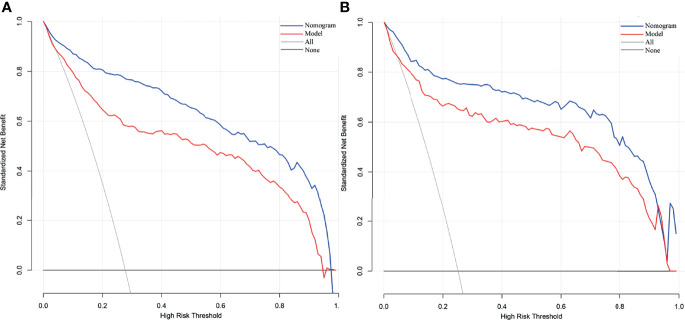
Decision curve analysis of our nomogram and model from development cohort **(A)** and validation cohort **(B)**. The net benefit versus the risk threshold probability is plotted. The x- and y-axes show the risk threshold probability and net benefit, respectively. A model is only clinically useful if it has a higher net benefit than the default treat-all and treat-none. It is clear from the graph that both the nomogram and model are superior to either treat-all or none strategy. Besides that, using the nomogram to distinguish ICC from HCC may get more benefit compared with model.

The independent difference factors between ICC and HCC were further used to establish the nomogram for ICC risk assessment (**Figure 2**). In addition, we also established the model incorporating AFP and CA199, which was commonly used at present in clinical. Compared with this model (AUC = 0.887; 95% CI, 0.865–0.910), the nomogram could distinguish ICC and HCC better with the AUC of 0.951 (95% CI, 0.938–0.964) (**Figure 3A**, *p* < 0.0001). In the validation cohort, compared with the model (AUC = 0.903, 95% CI: 0.865~0.942), the nomogram displayed the higher AUC of 0.958 (95% CI, 0.938–0.978) for the differentiation of ICC and HCC (**Figure 3B**, *p =* 0.0026). Diagnostic efficacies of the nomogram and compared model for distinguishing between ICC and HCC are listed in **Table 4**. After integrating patient preferences into the analysis, DCA displayed that both the nomogram and model would offer net benefits over the “treat-all” or “treat-none.” Upon further investigation, using this nomogram to distinguish ICC from HCC showed greater benefit when compared to the former model (**Figures 4A, B** **)**.

**Table 4:** Diagnostic efficacies of the nomogram and compared model for distinguishing between ICC and HCC.

Group	Variables	Nomogram	Model
Training cohort	AUC (95% CI)	0.951 (0.938–0.964)	0.887 (0.865–0.910)
	Sensitivity	85.24%	70.78%
	Specificity	92.72%	91.56%
	NPV	94.24%	89.09%
	PPV	81.79%	76.30%
Validation cohort	AUC (95% CI)	0.958 (0.938–0.978)	0.903 (0.865–0.942)
	Sensitivity	84.85%	78.79%
	Specificity	93.90%	89.49%
	NPV	94.86%	92.63%
	PPV	82.35%	71.56%

Nomogram consists of HBV, Log AFP, Log CA199, Log CA125, and Log PIVKA-II; model includes Log AFP and Log CA199.

AUC, area under the receiver operating characteristic curve; NPV, negative predictive value; PPV, positive predictive value; AFP, α-fetoprotein level; CA199, carbohydrate antigen 199; PIVKA-II, protein induced by vitamin K absence or antagonist-II; CA125, carbohydrate antigen 125; HBV, hepatitis B virus.

## Discussion

ICC has higher malignancy and poorer prognosis than HCC, whose morbidity and mortality rates have the tendency of heightening in recent years. Several etiological danger factors and clinical features of ICC and HCC are consistent with each other. However, it is widely accepted that resection of ICC is the only chance for cure,while the treatments for HCC patients include liver transplant, resection, radiofrequency ablation (RFA) and vascular interventional. Thus, histological analyses of tumor biopsies are required to discriminate ICC from HCC, which is important to provide appropriate treatment strategies as shown in international guidelines (5, 23). However, tumor biopsy is not allowed in most cases due to the advanced stage of disease and concomitant risks. Therefore, there is an urgent need for the accurate noninvasive way to correctly distinguish ICC and HCC (24–26).

AFP is a glycoprotein whose elevation is usually related to HCC. However, it does not increase significantly in about 35%–40% of HCC patients, especially for patients with small hepatocellular carcinoma (27, 28). PIVKA-II, also called des-γ-carboxyprothrombin (DCP), has been regarded as the ideal biomarker for the diagnosis and evaluation of HCC in recent years (28, 29). CA199 is a glycoprotein macromolecule that has been used as the marker in digestive system tumors and hepatobiliary disease (30, 31). CA125 is a mucin-type glycoprotein, sometimes named cancer antigen 125, produced by the mucin 16 (MUC16) gene (32). AFP and CA199 are often used to distinguish ICC and HCC. The results, however, have been unsatisfactory. Nomograms can be used for accurate assessment and identification of diseases, which provides a more simple but highly effective method for disease diagnosis and prognostic evaluation (33–35). The aim of our study was to establish such an efficient diagnostic nomogram model for clinical differentiation between ICC and HCC based on demographic characteristics and the routine laboratory tests. In this study, we found that HBV, Log AFP, Log CA199, Log CA125, and Log PIVKA-II were the independent elements of differentiation between them through the univariable and multivariable logistic regression. Based on these independent difference factors, we established a nomogram which displayed high accuracy by the AUC of 0.951 and 0.958 in the training and validation groups, respectively. Among those factors, HBV, Log AFP, and Log PIVKA-II were negatively correlated to ICC, while Log CA199 and Log CA125 were the positive factors for ICC. It is well known that the main danger factors for HCC are chronic infection with HBV or HCV, aflatoxin-contaminated foods, and so on. Furthermore, HBV infection is likely the predominant determinant of HCC in China (1). High levels of AFP and PIVKA-II are more common in HCC than ICC, the opposite of the CA199 and CA125.

The traditional model including AFP and CA199 was used as control. Compared to the control model (AUC = 0.887), the AUC of our nomogram was better at 0.951. This role of our nomogram was confirmed by the validation group with the AUC of 0.958. Previously, nomogram was evaluated using ROC and AUC, which lacked of the evaluation of clinical value. DCA could compensate for this deficiency, which is an effective method to assess the clinical benefits (36–38). The results of DCA showed that more benefits were increased through making use of our nomogram to differentiate ICC from HCC compared to the control model. Several models have been put forward to distinguish ICC from HCC. A nomogram combining six serum N-glycans was established for discriminating between ICC and HCC by Huang and collaborators. However, they found that the diagnostic performance of the nomogram might be better for those with poor liver function (39). Comprehensive analysis of metabolomics of ICC and HCC have been reported in recent years. Banales et al. (5) developed a nomogram based on serum metabolites (such as amino acids and sphingomyelins) that provided high values to distinguish patients with ICC from those with HCC. However, the detection of metabolites required special instruments and equipment, and the process was complex, which limited their clinical applications. Wang et al. (3) proposed a nomogram integrating six preoperative variables (gender, HBsAg, AST, AFP, CEA, and CA199) to discriminate ICC from HCC. AFP and CA199 were the independent difference factors between HCC and ICC, which was consistent with our research conclusion. However, the six variables of the nomogram were all categorical variables, which were difficult to quantify. The continuous variables included in our nomogram have been analyzed after logarithmic transformation, which can facilitate quantitative statistics. Furthermore, our nomogram recruits PIVKA-II, regarded as a better marker for HCC diagnosis that greatly improves the differential diagnostic capability between ICC and HCC. Above all, variables of our nomogram were preoperative routine tests, providing possible evidence for clinical application extensively. Nevertheless, some limitations of our study should be recognized. The data of this study were obtained from a single institution; thus, external and large samples are needed for further validation. In addition, since variables of our nomogram were closely related with the differentiation of liver cancer, the diagnostic value of the nomogram might be better for those with poor differentiation.

## Conclusion

In conclusion, we demonstrated that HBV, Log AFP, Log CA199, Log CA125, and Log PIVKA-II were the independent differential factors between ICC and HCC. The nomogram incorporating these five commonly assessed preoperative factors was established for optimal discrimination between them. Further application of this nomogram in multicenter investigations may confirm its clinical value.

## Data Availability Statement

The original contributions presented in the study are included in the article/supplementary material. Further inquiries can be directed to the corresponding authors.

## Ethics Statement

The studies involving human participants were reviewed and approved by Ethics Committee of the Shandong Provincial Hospital Affiliated to Shandong First Medical University. The data were anonymous, and the requirement for informed consent was therefore waived.

## Author Contributions

ZL and YW conceived the project and designed the experiments. CP performed the data extraction and analyzation. YS and XW wrote and revised the manuscript. All authors contributed to the article and approved the submitted version.

## Funding

This work was supported by the grants from the Medicine and Health Science and Technology Development Plan of Shandong Province (No. 2019WS474 http://sdkyxm.wsglw.net/).

## Conflict of Interest

The authors declare that the research was conducted in the absence of any commercial or financial relationships that could be construed as a potential conflict of interest.

## Publisher’s Note

All claims expressed in this article are solely those of the authors and do not necessarily represent those of their affiliated organizations, or those of the publisher, the editors and the reviewers. Any product that may be evaluated in this article, or claim that may be made by its manufacturer, is not guaranteed or endorsed by the publisher.
